# Dissociation Between Subjective Fatigue and Objective Performance During Repeated Tackling in Amateur Rugby Players

**DOI:** 10.1155/tsm2/5566824

**Published:** 2025-11-30

**Authors:** Yuto Kikuchi, Kiyokazu Akasaka, Yutaka Sawada, Yu Okubo, Hiroshi Hattori, Yuji Hamada, Toby Hall

**Affiliations:** ^1^Graduate School of Medicine, Saitama Medical University, Moroyama, Saitama, Japan; ^2^Department of Rehabilitation, Kawagoe Clinic, Saitama Medical University, Kawagoe, Saitama, Japan; ^3^School of Physical Therapy, Saitama Medical University, Moroyama, Saitama, Japan; ^4^Curtin School of Allied Health, Curtin University, Perth, Western Australia, Australia

**Keywords:** countermovement jump, fatigue-performance dissociation, kinematic analysis, rugby tackling, subjective fatigue

## Abstract

**Background:**

Rugby involves frequent, high-intensity collisions. While cumulative fatigue is known to impair performance, recent evidence suggests a dissociation between subjective fatigue and objective performance. Understanding this gap is critical for optimizing training and reducing injury risk in collision sports.

**Methods:**

Twenty-nine amateur male rugby players each performed 30 maximal-effort tackles against a stationary tackle dummy. The kinetics and kinematics tackle motion was captured using a 3D motion analysis system (Vicon Motion Systems Ltd, UK) including 10 motion capture cameras. The subjective fatigue, encompassing physical, mental, and motivation aspects, was assessed using visual analog scales at four distinct time points (Pre, Post1, Post2, and Post3). The objective performance was evaluated through countermovement jump (CMJ) height and tackle variables (velocity, momentum, and kinetic energy) extracted from the tackle motion. The analyses included repeated-measures ANOVA, Friedman tests, linear mixed models, and Spearman's correlations.

**Results:**

Physical and mental fatigue increased significantly over time (*p* < 0.05), particularly after the 20th tackle. However, despite a slight improvement in CMJ performance, no statistically significant changes were detected in kinetics and kinematics parameters. A moderate positive association was revealed between CMJ height and tackle velocity, as determined by correlational analysis. However, no significant relationship was found between subjective fatigue and CMJ height.

**Conclusion:**

Repeated tackling led to substantial subjective fatigue without corresponding declines in objective performance. This dissociation suggests that athletes may experience fatigue without associated actual performance impairment. Perceived fatigue increased markedly after around 20 tackles. However, performance decline may occur only beyond this threshold, which was not reached in the present protocol. These results highlight the need to integrate subjective and objective assessments when monitoring athletes to optimize training and reduce injury risk.

## 1. Introduction

Rugby football, also known as rugby, is a sport that is defined by its frequent and high-intensity physical contact, which is characterized by high impact collisions between players [1, 2]. In the course of competition, athletes engage in a series of high-demand movements, including tackling, sprinting, stepping, changing direction, and jumping [3, 4]. Among the aforementioned actions, tackling is a fundamental and frequently executed skill, particularly by defensive players who aim to impede the progress of the offense [5]. Rennie et al. found that the mean number of tackles per match increased substantially in the 2020 season compared to the 2014–2019 seasons [6].

Tackles are also closely associated with injury incidence in rugby. A substantial body of research has previously indicated that a considerable percentage of injuries are sustained by tackling players, with a notable occurrence in the latter phases of sporting events [7–10]. This heightened injury risk during the latter half of the competition can be attributed to the cumulative effects of both physical and mental fatigue associated with repeated tackling and other high-intensity actions such as sprinting and direction changes.

A multitude of studies have demonstrated that physical fatigue impairs muscular strength and reduces movement accuracy [11–13]. Concurrently, mental fatigue leads to declines in concentration and attention [14], collectively contributing to decreased performance. However, the relationship between fatigue and performance is not always linear. Recent studies have indicated a possible discrepancy between self-reported fatigue and objective performance outcomes [15]. For instance, athletes may report elevated levels of fatigue without demonstrating significant declines in objective performance metrics, such as jump height [16]. This phenomenon is believed to be influenced by psychological factors, motivation, and skill automation, highlighting the importance of utilizing both subjective and objective assessments to comprehensively evaluate an athlete's condition [14].

Despite the plethora of studies that have examined the relationship between fatigue and performance, research specifically examining this relationship in the context of consecutive tackles in rugby remains limited. Furthermore, numerous preceding studies have conducted field-based measurements subsequent to actual matches, which are inherently subject to extraneous factors such as weather conditions, interpersonal interactions, and the dynamics of gameplay. Conversely, laboratory-based studies, such as the present one, permit greater control over extraneous variables by standardizing tackle execution. This, in turn, enables more reproducible assessments of fatigue and performance changes.

The objective of this study was to investigate the effects of repeated tackle efforts on both subjective fatigue and objective performance among amateur rugby players and to explore the relationship between these parameters. The hypothesis of this study was that repeated tackles would lead to increased physical and mental fatigue and decreased performance, thereby contributing to a higher risk of injury.

## 2. Methods

### 2.1. Participants

Twenty-nine healthy amateur male rugby players participated in this study. The required sample size was estimated using G∗Power 3.1 (http://www.gpower.hhu.de/).

Assuming a medium effect size (*f* = 0.25), *α* = 0.05, and power (1 − *β*) = 0.80 for a repeated-measures ANOVA, the calculation indicated that 24 participants were required. To account for potential attrition, the target recruitment was set at 30 players. Exclusion criteria included refusal to provide informed consent, injury preventing tackle performance, ongoing medical or surgical treatment, or a history of concussion with prolonged recovery (> 21 days). Prior to participation, all subjects were informed of the study's objectives and procedures and provided written informed consent. The study was approved by the Ethics Committee of Saitama Medical University (Approval No. 2024-008).

Prior to testing, participants were instructed to adhere to the following guidelines for at least 24 h before the testing session: (1) obtain a minimum of 7 h of sleep, (2) consume 35 mL of water per kilogram of body weight, (3) abstain from alcohol consumption, and (4) refrain from high-intensity exercise on the day before testing. On the day of testing, participants were required to (1) abstain from caffeine and nicotine for a minimum of 3 h prior to testing and (2) consume only a light meal at least 2 hours before testing [16]. It is noteworthy that all participants adhered to the stipulated instructions.

### 2.2. Motion Analysis

To assess body movements during tackles, a three-dimensional motion capture system (Vicon Vero 2.2, Vicon Motion Systems, Oxford, UK) with 10 infrared cameras was utilized at a sampling rate of 250 Hz. The data were processed using Vicon Nexus 2.12.1 software. A 15-Hz low-pass Butterworth filter was applied to the kinematic data to remove high-frequency noise [17]. During the measurement phase, the participants were outfitted with a head cap, mouthguard, compression shorts, and indoor sports shoes.

The placement of reflective markers followed the guidelines of the Plug-in Gait model. To facilitate comprehensive spinal motion analysis, an additional set of 10 markers was attached to the subject's spine. Markers were attached to the following structures: the spinous process of the third thoracic vertebra (T3), the bilateral transverse processes of the seventh thoracic vertebra (T7), the spinous process of the 12th thoracic vertebra (T12), and the bilateral transverse processes of the first lumbar vertebra (L1). Additional marker points were the spinous processes of the third lumbar vertebra (L3), the bilateral transverse processes of the fourth lumbar vertebra (L4), and the spinous processes of the fifth lumbar vertebra (L5), which were identified and used as the anatomical landmarks for reflective marker placement in this case study [18].

Prior to the start of testing, all participants underwent a 10-min warm-up procedure involving the use of a cycle ergometer. Subsequently, they executed a maximal-effort tackle at a height representative of the game against a tackle dummy (SUZUKI RUGBY Hard Tackle Dummy SD-903) positioned at a distance of 4.5 m. The initial set of three tackles was performed (Pre), followed by a 10-min rest period. Subsequently, the participants were engaged in a series of additional tackles, continuing until a total of 30 had been attained. The collection of kinematic data occurred during Tackles 8–10 (Post1), 18–20 (Post2), and 28–30 (Post3). Each tackle was executed at two-minute intervals.

For each time point, the mean value of the three tackle trials was used for analysis. In cases where one or more trials were excluded due to marker dropout or acquisition failure, the mean of the remaining valid trials was used to represent that measurement point. In the event that all three trials were missing, the corresponding data were considered missing. To ensure maximal effort, trials in which tackle speed fell below 70% of the individual's preaverage were excluded from the analysis.

### 2.3. Experimental Design

Assessments were conducted at four time points (Pre, Post1, Post2, and Post3).

At Pre, participants first completed the VAS assessment, then performed three tackles, followed by the countermovement jump (CMJ). For Post1–Post3, participants performed 10 tackles for each set, after which they completed the VAS assessment and then the CMJ. This sequence was applied consistently to monitor changes in subjective fatigue and objective performance across the repeated tackle protocol.

### 2.4. Outcome Measures

#### 2.4.1. Subjective Measures

The subjective experience of fatigue was measured using a visual analog scale (VAS). The following three items were evaluated at each time point (Pre, Post1, Post2, and Post3). The factors under consideration were physical fatigue, mental fatigue, and motivation. Each of these three factors was evaluated separately using a 100-mm VAS. For the fatigue assessments, participants rated both physical and mental fatigue independently, with 0 mm indicating “no fatigue” and 100 mm indicating “maximum fatigue.” Motivation was assessed using a separate VAS, where 0 mm indicated “no motivation” and 100 mm indicated “maximum motivation.” The left endpoint of the scale indicated a complete absence of motivation, while the right endpoint represented the maximum level of motivation. Participants completed the VAS independently at each time point using a new sheet to prevent reference to prior responses.

#### 2.4.2. Kinetics and Kinematics Parameters

The objective performance of the subjects was assessed using three kinetics and kinematics parameters derived from tackling: velocity, momentum, and kinetic energy. Velocity was calculated by dividing the displacement of the pelvis center over successive frames by the elapsed time [19]. The mean velocity over the 0.1-s interval (25 frames) immediately preceding contact with the tackle dummy was utilized for the analysis. Momentum (*P*, kg·m/s) was computed as *P* = *m* × *v*, and kinetic energy (KE, J) as KE = 1/2 × *m* × *v*^2^ [1].

#### 2.4.3. CMJ

Lower-limb performance was assessed using the CMJ. Flight time was measured using a motion analysis system (Vicon Vero 2.2, Vicon Motion Systems Ltd., Oxford, UK). Participants began each jump from an upright stance with their feet shoulder-width apart, squatted to approximately 90° of knee flexion, and performed a maximal vertical jump using both legs equally. Participants kept their hands fixed on their hips throughout the jump to eliminate the influence of arm swing. CMJ was performed at four time points (Pre, Post1, Post2, and Post3), and the mean value of three trials at each time point was used for the analysis. Jump height (cm) was calculated from flight time (*t*, *s*) using the equation: *h* = 1/8 × *g* × *t*^2^, where *g* is 9.81 m/s^2^.

### 2.5. Statistical Analysis

All statistical analyses were conducted using IBM SPSS Statistics Version 29 (IBM Corp., Armonk, NY, USA). Initially, the normality of the data was assessed. For non-normally distributed subjective variables (i.e., physical fatigue, mental fatigue, and motivation), the Friedman test was used to evaluate changes over time. For CMJ, which was distributed in a normal manner, a repeated-measures ANOVA was performed. In instances where a substantial main effect was identified, post hoc comparisons were executed with the aid of the Bonferroni correction.

The kinetics and kinematics parameters (i.e., velocity, momentum, and kinetic energy) contained some missing data; therefore, a linear mixed model was employed to examine the main effects over time. If necessary, a Bonferroni correction was applied for multiple comparisons. To explore associations between subjective measures, kinetics and kinematics parameters, and CMJ at each time point, Spearman's rank correlation coefficients were calculated. The significance level for all tests was set at *p* < 0.05.

## 3. Results

A total of 29 healthy amateur male rugby players participated in this study. The participants had a mean age of 23.7 ± 3.4 years, height of 171.6 ± 4.7 cm, body mass of 73.7 ± 11.7 kg, and an average rugby experience of 7.4 ± 5.4 years. A total of 348 tackles were executed during the study, of which 321 (92%) were successfully analyzed.

An analysis was conducted to examine temporal changes in subjective fatigue and objective performance at each measurement time point (see Table 1). With regard to the kinetics and kinematics parameters (i.e., velocity, momentum, and kinetic energy), no statistically significant main effects were observed over time (*p* > 0.05 for all). Conversely, a substantial main effect was identified for CMJ height (*p* < 0.05). However, Bonferroni-adjusted post hoc comparisons revealed that there were no statistically significant differences between measurement time points (*p* > 0.05).

Both physical and mental fatigue scores increased significantly over time (*p* < 0.05). Post hoc comparisons revealed significant differences between Pre and Post2, Pre and Post3, and Post1 and Post3 for both measures, indicating a progressive increase in perceived fatigue across repeated tackles. No significant difference was observed between Pre and Post1, and motivation scores remained unchanged across all time points (*p* > 0.05).

To examine the relationships between subjective fatigue and objective performance at each measurement time point, Spearman's rank correlation coefficients were calculated (Table 2). The findings indicated a moderate positive correlation between CMJ height and tackle velocity at all time points, with a statistical significance level of *p* < 0.01. Conversely, no substantial correlations were identified between subjective fatigue measures (i.e., physical fatigue, mental fatigue, and motivation) and CMJ at any given time point (*p* > 0.05). Furthermore, a weak negative correlation was observed between physical fatigue and momentum at the Pre time point only (*p* < 0.05).

## 4. Discussion

The present study examined the impact of repeated tackling on subjective fatigue and objective performance among amateur rugby players. The findings indicated that both subjective physical fatigue and mental fatigue ratings increased significantly with repeated tackles. However, these increases were not accompanied by measurable declines in objective performance, suggesting a dissociation between perceived fatigue and actual physical capacity. Conversely, while CMJ performance exhibited a tendency toward enhancement over time, no substantial variations were identified following Bonferroni-adjusted multiple comparisons at the subsequent time points. While weak correlations were observed between certain subjective ratings and kinetics and kinematics parameters, the majority of relationships did not reach statistical significance. Notably, both physical and mental fatigue increased over time, while CMJ showed a slight upward trend. This finding contradicts our initial hypothesis, which proposed that accumulated fatigue from repeated tackles would lead to performance decline. The results suggest a potential dissociation between perceived fatigue and actual physical capacity. While fatigue is generally associated with decreased muscle strength, power output, and movement accuracy [20, 21], the results of this study demonstrated increased fatigue without a corresponding decline in either CMJ or tackle performance. Both CMJ height and tackle-related kinetic variables (velocity, momentum, and kinetic energy) remained stable throughout the protocol, indicating that athletes maintained consistent tackle execution despite increasing subjective fatigue. Interestingly, although the exercise protocol did not induce measurable declines in physical performance, significant increases in mental fatigue were observed. This may suggest that mental fatigue could precede physical performance decline, serving as a potential early indicator of reduced performance capacity during prolonged or high-intensity efforts. Discrepancies between subjective reports of fatigue and objective performance measures have been documented in other sports as well, and the present findings lend further support to this notion [21, 22].

However, maintained CMJ performance does not imply absence of fatigue. Conversely, an increase in subjective fatigue has the potential to compromise the quality and safety of tackling by diminishing attention or decision-making capacity. Previous studies have demonstrated that tackling technique deteriorates under fatigue [3, 23] and that injury incidence increases during the second half of matches [24–26]. In these studies, fatigue primarily referred to physiological or physical fatigue induced by repeated high-intensity efforts, rather than self-reported subjective fatigue. These findings underscore the importance of combining objective performance metrics with subjective assessments to accurately evaluate athletes' physical and psychological conditions. From a pragmatic standpoint, an integrated monitoring approach that incorporates both aspects is important for effective player management in competitive environments.

A notable finding was the stability in motivation levels across the study period. One potential explanation for this phenomenon is that the nature of the task did not impose significant mental strain or require prolonged cognitive engagement. The task consisted of a standardized tackle protocol with intertrial rest periods, which may have reduced the overall demand on the subjects. Furthermore, the players' familiarity with the task may have mitigated significant fluctuations in their motivational state. A growing body of research has demonstrated that mental fatigue impedes physical performance, primarily by increasing perceived exertion, particularly during endurance activities [27]. Conversely, the brief, intermittent nature of the present protocol may not have elicited a commensurate effect on motivation. Furthermore, motivation is influenced by complex psychological constructs, including task meaning and achievement goals [28]. It is conceivable that, given the discrepancy between the protocol's design and real game or training scenarios, participants were able to sustain a consistent level of intrinsic motivation, which remained unaltered despite the rise in physical and mental fatigue.

The finding that subjective fatigue levels increased significantly after approximately 20 tackle repetitions has practical implications for designing effective training feedback and intervention strategies. The manifestation of fatigue, which is evident to the observer, beyond a certain threshold, underscores the significance of prompt coaching interventions. Such interventions may include technical feedback, rest periods, and psychological support. The purpose of these interventions is to avert declines in concentration and technical proficiency. In high-intensity training sessions or contact-heavy drills, immediate interventions based on early signs of fatigue may help maintain learning outcomes and competitive performance, while also contributing to injury prevention [29, 30].

### 4.1. Limitation

It is important to acknowledge the limitations of this study. Firstly, the physical load applied during the protocol may have been comparatively low when compared to the actual match or training conditions. The 2-min rest intervals between trials may have mitigated fatigue accumulation. In real-game scenarios, tackles frequently occur in rapid succession and under circumstances that are not easily predicted. In contrast, the present study was conducted in a university laboratory setting and involved unidirectional tackles against a static dummy. Consequently, the protocol may not have fully replicated the physical and cognitive demands of match play.

Secondly, the participant pool comprised exclusively of amateur male rugby players, which may limit the generalizability of the findings. The degree of fatigue accumulation and its impact on performance may vary depending on factors such as competitive level, age, and sex. Therefore, it is important to exercise caution when applying these results to other athletic populations.

## 5. Conclusion

Repeated tackling led to a significant increase in subjective fatigue, while no clear decline was observed in objective performance measures, including both CMJ and tackle-related kinetic variables. These findings suggest a potential dissociation between perceived fatigue and physical capacity. A notable finding was the marked increase in subjective fatigue after approximately 20 tackles, which offers valuable insight into the optimal timing for coaching interventions and rest implementation during training. In order to accurately assess player condition and promote both performance maintenance and injury prevention, it is essential to establish a monitoring system that integrates both subjective and objective evaluation methods.

## Figures and Tables

**Table 1:** Temporal changes and multiple comparisons of subjective fatigue and performance metrics.

Variable	Pre	Post1	Post2	Post3	*p* value	Significant pairwise comparisons
Physical fatigue (mm)	18.2 ± 22.0	38.0 ± 22.3	49.7 ± 24.1	61.9 ± 24.6	< 0.001	Pre < Post2
						Pre < Post3
						Post1 < Post3

Mental fatigue (mm)	16.9 ± 23.6	24.9 ± 23.4	35.2 ± 29.1	39.3 ± 32.8	< 0.001	Pre < Post2
						Pre < Post3
						Post1 < Post3

Motivation (mm)	71.1 ± 28.4	77.6 ± 19.7	78.5 ± 21.3	75.3 ± 24.5	0.23	None

CMJ height (cm)	41.0 ± 6.5	41.7 ± 6.3	41.8 ± 6.8	42.4 ± 7.3	0.03	None

Tackle velocity (m/s)	4.1 ± 0.5	4.2 ± 0.5	4.1 ± 0.5	4.4 ± 0.5	0.25	None

Momentum (kg·m/s)	368.6 ± 60.5	375.8 ± 60.7	375.6 ± 52.4	375.8 ± 60.4	0.30	None

Kinetic energy (J)	929.1 ± 199.2	966.8 ± 212.7	954.9 ± 189.5	975.3 ± 214.8	0.27	None

*Note:* Physical fatigue, mental fatigue, motivation, and CMJ were assessed after the 10th, 20th, and 30th tackles. Tackle velocity, momentum, and kinetic energy were calculated based on Trials 8–10, 18–20, and 28–30, respectively. When trials were invalid due to marker dropout or acquisition error, the mean of the remaining valid trials was used. Data points were excluded if all trials were missing. Values are presented as mean ± SD.

Abbreviation: CMJ, countermovement jump.

**Table 2:** Correlations between subjective fatigue, mechanical parameters, and CMJ across time points.

Combination	Pre	Post1	Post2	Post3
Physical fatigue × speed	0.00	0.17	0.14	−0.05
Physical fatigue × momentum	−0.39^∗^	−0.24	−0.30	−0.30
Physical fatigue × kinetic energy	−0.22	−0.06	−0.11	−0.16
Physical fatigue × CMJ	−0.14	0.08	−0.09	−0.13
Mental fatigue × speed	0.22	0.14	0.10	−0.15
Mental fatigue × momentum	−0.07	−0.09	−0.03	−0.04
Mental fatigue × kinetic energy	0.09	0.04	0.11	−0.04
Mental fatigue × CMJ	−0.06	−0.05	−0.16	−0.15
Motivation × speed	−0.12	−0.06	−0.06	0.07
Motivation × momentum	−0.03	−0.06	−0.25	−0.12
Motivation × kinetic energy	−0.14	−0.08	−0.21	−0.13
Motivation × CMJ	−0.02	−0.05	0.07	0.12
CMJ × speed	0.53^∗∗^	0.52^∗∗^	0.49^∗∗^	0.55^∗∗^
CMJ × momentum	0.13	0.14	0.14	0.10
CMJ × kinetic energy	0.35	0.27	0.36	0.37

*Note:* Physical fatigue, mental fatigue, motivation, and CMJ were assessed after the 10th, 20th, and 30th tackles. Tackle velocity, momentum, and kinetic energy were calculated based on Trials 8–10, 18–20, and 28–30, respectively. When certain trials were invalid due to marker dropout or acquisition error, the mean of the remaining valid trials was used. Spearman's rank correlation coefficients (*ρ*) are shown.

Abbreviation: CMJ, countermovement jump.

^∗^ *p* < 0.05 and ^∗∗^*p* < 0.01.

## Data Availability

The data that support the findings of this study are available upon request from the corresponding author. The data are not publicly available due to privacy or ethical restrictions.
